# A holistic approach to maximise diagnostic output in trio exome sequencing

**DOI:** 10.3389/fped.2023.1183891

**Published:** 2023-05-19

**Authors:** Sandra von Hardenberg, Hannah Wallaschek, Chen Du, Gunnar Schmidt, Bernd Auber

**Affiliations:** Department of Human Genetics, Hannover Medical School, Hannover, Germany

**Keywords:** trioWES, CNV, GeneMatcher, re-evaluation, diagnostic odyssey

## Abstract

**Introduction:**

Rare genetic diseases are a major cause for severe illness in children. Whole exome sequencing (WES) is a powerful tool for identifying genetic causes of rare diseases. For a better and faster assessment of the vast number of variants that are identified in the index patient in WES, parental sequencing can be applied (“trio WES”).

**Methods:**

We assessed the diagnostic rate of routine trio WES including analysis of copy number variants in 224 pediatric patients during an evaluation period of three years.

**Results:**

Trio WES provided a diagnosis in 67 (30%) of all 224 analysed children. The turnaround time of trio WES analysis has been reduced significantly from 41 days in 2019 to 23 days in 2021. Copy number variants could be identified to be causative in 10 cases (4.5%), underlying the importance of copy number variant analysis. Variants in three genes which were previously not associated with a clinical condition (*GAD1*, *TMEM222* and *ZNFX1*) were identified using the matching tool GeneMatcher and were part of the first description of a new syndrome.

**Discussion:**

Trio WES has proven to have a high diagnostic yield and to shorten the process of identifying the correct diagnosis in paediatric patients. Re-evaluation of all 224 trio WES 1–3 years after initial analysis did not establish new diagnoses. Initiating (trio) WES as a first-tier diagnostics including copy number variant detection should be considered as early as possible, especially for children treated in ICU, if a monogenetic disease is suspected.

## 1. Introduction

Rare genetic disorders often manifest as severe multisystemic disorders and represent a significant cause of pediatric hospitalisation (1–3). Studies show that 21%–57% of children admitted to an intensive care unit (ICU) suffer from a genetic disorder (4–6). In a recent study, monogenetic diseases were identified as causative in 41% of infant deaths. Treatment options, that have the potential to improve the clinical outcome, would have been available for 30% of these diseases (7). Consequently, several studies have shown that children with suspected monogenetic disorders benefit from a fast and precise diagnosis to ensure rapid and efficient personalized treatment (8, 9). Recently, next generation sequencing (NGS) technologies such as whole exome sequencing (WES) or whole genome sequencing (WGS) have become more accessible in terms of cost and turnaround time (TAT) and are increasingly used to identify the underlying genetic causes of rare diseases. Simultaneous analysis of the patient and their parents [trio WES (tWES)] has the potential to speed up the process of identifying the correct genetic diagnosis even more (10). In particular, severely ill infants who are admitted to neonatal or paediatric intensive care units (NICU/PICU) benefit from access to rapid, extensive genetic diagnostics leading to precision treatments (9). This in turn leads not only to reduced costs of care but also shortens the often very long time (“diagnostic odyssey”) from the clinical description of an unidentified rare disease to a definitive diagnosis (11). The large majority of parents and clinicians agreed that genomic results were beneficial and had a very high perceived utility even when testing did not yield a diagnosis (12, 13).

However, in many countries, the implementation of broad genetic testing has not been established in routine diagnostics yet and coverage by health insurances still remains a challenge for WES or WGS and especially for trio analysis (6, 14–16).

The aim of this study is to demonstrate how a holistic, multimodal approach that includes patient´s accurate clinical information, the use of parental information, a comprehensive analytic and clinical variant assessment, and the use of GeneMatcher (17) can maximize the chance of establishing a genetic diagnosis in routine diagnostics.

## 2. Methods and materials

### 2.1. Patient cohort

The study was conducted in accordance with the general principles outlined in the Declaration of Helsinki (18) and approved by the local Institutional Review Board of Hanover Medical School (ID 8657_BO_K_2019). The analyzed cohort included 224 index patients and their unaffected parents of different ethnicity with a suspected genetic condition who were referred to our laboratory for genetic testing between the years 2019 and 2021.

### 2.2. Sample preparation for whole exome sequencing

DNA was extracted from blood using NucleoMag Blood kit (Macherey-Nagel, Germany). Exome sequencing was performed using the IDT Exome (xGen Exome V02, IDT, Leuven, Belgium). The captured DNA was sequenced using Illumina NextSeq 500, Illumina NextSeq550, NovaSeq 6000 or MGI DNB SEQ G400RS Sequencer to generate 150-bp paired-end reads. Reads were aligned to the human reference genome (UCSC Genome Browser build GRCh37/hg19). Sequence data were processed using the megSAP analysis pipeline (https://github.com/imgag/megSAP), regarding quality control, read alignment and variant annotation/detection. To verify the DNA sample, 14 single nucleotide polymorphisms (SNP) were amplified by competitive allele-specific PCR using fluorescence-labeled primers and analysed by StepOnePlus software for genotyping experiments (StepOnePlus System, Thermo Fischer Scientific, Inc., Waltham, MA, USA). SNP results were compared with the data from the NGS analysis (megSAP). Reads were aligned to the human reference genome (UCSC Genome Browser build GRCh37).

### 2.3. Sequence variant filter strategy

Sequence data were analyzed using GSvar (https://github.com/imgag/ngs-bits) and IGV (http://software.broadinstitute.org/software/igv/). All quality metrics were examined prior to analyzation of data. Default cut-offs were used for target region read depth (20x coverage), target region 20x percentage (above 95%), known variants percentage (above 98%) and SNV allele frequency deviation (indicative of DNA contamination, must be below 3%). The aforementioned QC thresholds were established using aggregated sample QC data (derived from >150 exome datasets). Variant filtering was applied to identify candidate disease-causing single nucleotide variants (SNVs) in the index patient using the following parameters: rare variants with allele frequency <1% in the overall population of the 1,000 Genomes Project and the genome Aggregation Database (gnomAD); variants that occur in our internal database less than 20 times; variants annotated as pathogenic (p) or likely pathogenic (lp) in ClinVar or the Human Gene Mutation Database (HGMD); homozygous or compound heterozygous variants, *de novo* variants or inherited X-chromosomally variants in male patients. Variants that were localised in the intron region beyond ±1–20 bases from the intron/exon border as well as variants with low mapping quality were excluded. Intronic variants at ±>20 bases from the intron/exon border were evaluated for pathogenicity if they were classified in ClinVar as p/lp.

### 2.4. Clinical interpretation and confirmation of variants

Variants which passed the filtering criteria, were evaluated using the ACMG/AMP (American College of Medical Genetics and Genomics/Association for Molecular Pathology) guidelines on variant interpretation and classification (19, 20) using the following terms: class 5 (pathogenic, p), class 4 (likely pathogenic, lp), class 3 (variants of unknown significance, VUS), class 2 (likely benign) and class 1 (benign). Variant nomenclature is based on standardized Human Genome Variation Society (HGVS) conventions as referenced in den Dunnen et al. (21). In addition to variant allele frequency data, we used prediction tools, including phyloP, SIFT, PolyPhen2, FATHMM, REVEL and CADD, and searched databases, including LOVD, ClinVar, and gnomAD. Furthermore, for each gene carrying a rare *de novo variant* or rare biallelic variants, we performed an extensive literature search and, if necessary, contacted the authors of publications. Additionally, all potentially p/lp variants in genes that are not associated with a specific phenotype and met the filter criteria, were submitted to GeneMatcher (17).

### 2.5. Secondary findings

The analysis of clinically actionable genetic variants that are unrelated to the primary indication for testing (secondary findings) was offered to each individual tested as part of trio-based analysis our in-house recommendations for the return of secondary findings (22). We performed a targeted search for (p/lp) variants in patients who consented to the receipt of secondary findings: In children, we reported p/lp variants in *APC*, *APOB*, *LDLR*, *MEN1*, MLH1*, MSH2*, MSH6*, *PCSK9*, PMS2*, *RET*, *TP53* and *VHL*. In adults, we reported p/lp variants in *APC*, *APOB*, *BRCA1*, *BRCA2*, *CDH1*, *LDLR*, *MEN1*, *MLH1*, *MSH2*, *MSH6*, MUTYH*, *PALB2*, *PCSK9*, PMS2*, *RET*, *TP53* and *VHL* [* = only homozygous or compound heterozygous p/lp variants (potentially biallelic)].

### 2.6. Copy number variants

Copy number variant (CNV) calling was performed using ClinCNV (23) based on the depth of coverage, i.e., the number of reads, in similar probes. In WES, exon target regions alternate with non-target intron regions. For each region, the expected depth of coverage is compared with the observed depth of coverage and a log-likelihood of a copy-number alteration is given for each region. The following sample-specific information of the CNV caller were used to assess variants: genomic position, size (kb), number of regions (exons), affected genes, copy-number change, log-likelihood (logarithm of the ratio between likelihoods of the no CN change model vs. the CN equal to the reported state model), potential allele frequency (AF, frequency of the copy-number change in the analysed cohort, i.e., the 100–200 most similar samples), *q*-value (*p*-value corrected for the number of CNVs detected). Rare copy number variants affecting OMIM genes were followed up similarly as SNV and small insertions and deletions (indels) (19) described above.

### 2.7. Submission to GeneMatcher

For non-synonymous, frameshift and nonsense variants or variants that affect donor/acceptor splice sites in genes of unknown significance (GUS) which were in homozygous or compound heterozygous state or represented as a *de novo* germline event in the patient, an intensive literature search was carried out. If an association between the GUS and the patient's disease was suspected, potential p/lp variants in GUS were shared through the international collaborative platform GeneMatcher to identify additional patients and to improve genotype–phenotype correlations (24). If a match was obtained, stakeholders were contacted by mail and the phenotypes were compared.

### 2.8. Re-evaluation of tWES data

Re-evaluation analysis of all tWES was carried out in November 2022 after a period of at least 1 year (range 1–3 years) after the last diagnostic test using GSVar and VarSeq® 2.2.4 software (Golden Helix). For this effort, annotations were renewed, variant filtration was repeated and databases LOVD, ClinVar, and gnomAD were re-searched. If new clinical data of the index patient was available, prioritised variants underwent further clinical-genetic assessment.

## 3. Results

### 3.1. Diagnostic testing results

224 children and adolescents (134 males and 90 females), ranging in age from 2 days to 18 years with a mean age of 3.7 years, suspected of having a monogenetic disease were tested in parallel with their parents (tWES) in a diagnostic setting over a period of 3 years (2019–2021). Disease phenotypes were highly diverse and categorized in 12 groups according to the clinical data provided by the referring physician (Table 1). Most patients had a suspected genetic immunodeficiency (*n* = 68; 30%), followed by patients with a complex disorder belonging to the spectrum of syndromic (*n* = 55; 25%) and neurologic disease (*n* = 42; 19%). Overall, a molecular diagnosis was provided for 67 patients [causative p/lp variant(s), 30%]. In 32 patients (14%), identification of a VUS resulted in an inconclusive report. In 125 patients (56%), no variants that were (obviously) related to the disease phenotype could be identified. The highest diagnostic rate was achieved in patients affected by syndromic disorders (*n* = 23/55, 42%) (Table 1). No relevant difference was observed when comparing the age of patients with or without molecular diagnosis (data not shown).

**Table 1:** Clinical presentation of the children at the time of the genetic analysis.

Disease group	Genetic diagnosis	Inconclusive genetic result	No genetic diagnosis	In total	% of total
Cardiology	2	3	5	10	4.4%
Dermatology	1	-	1	2	0.9%
Hepatopathy	2	1	3	6	2.7%
Immunodeficiency	15	10	43	68	30.4%
Nephropathy	2	1	1	4	1.8%
Metabolic disorder	4	2	2	8	3.6%
Mitochondriopathy	1	-	-	1	0.4%
Myopathy	2	1	-	3	1.3%
Neurology	13	3	26	42	18.8%
Oncology	-	1	7	8	3.6%
Pulmology	2	3	11	16	7.1%
Syndromology	23	7	25	55	24.6%
Vasculopathy	-	-	1	1	0.4%
In total	67	32	125	224	100%

Classification of the disease of the patients in different upper groups. The number of children who received a genetic diagnosis (class 4 and class 5 variants) are classified under “Genetic diagnosis”. Children who received an inconclusive result [class 3 variants (VUS)] are classified under “Inconclusive genetic result”. Children who received no result (class 1 and class 2 variants) are classified under “No genetic diagnosis”. “In total” presents the total number of children in each group.

**Figure 1:**
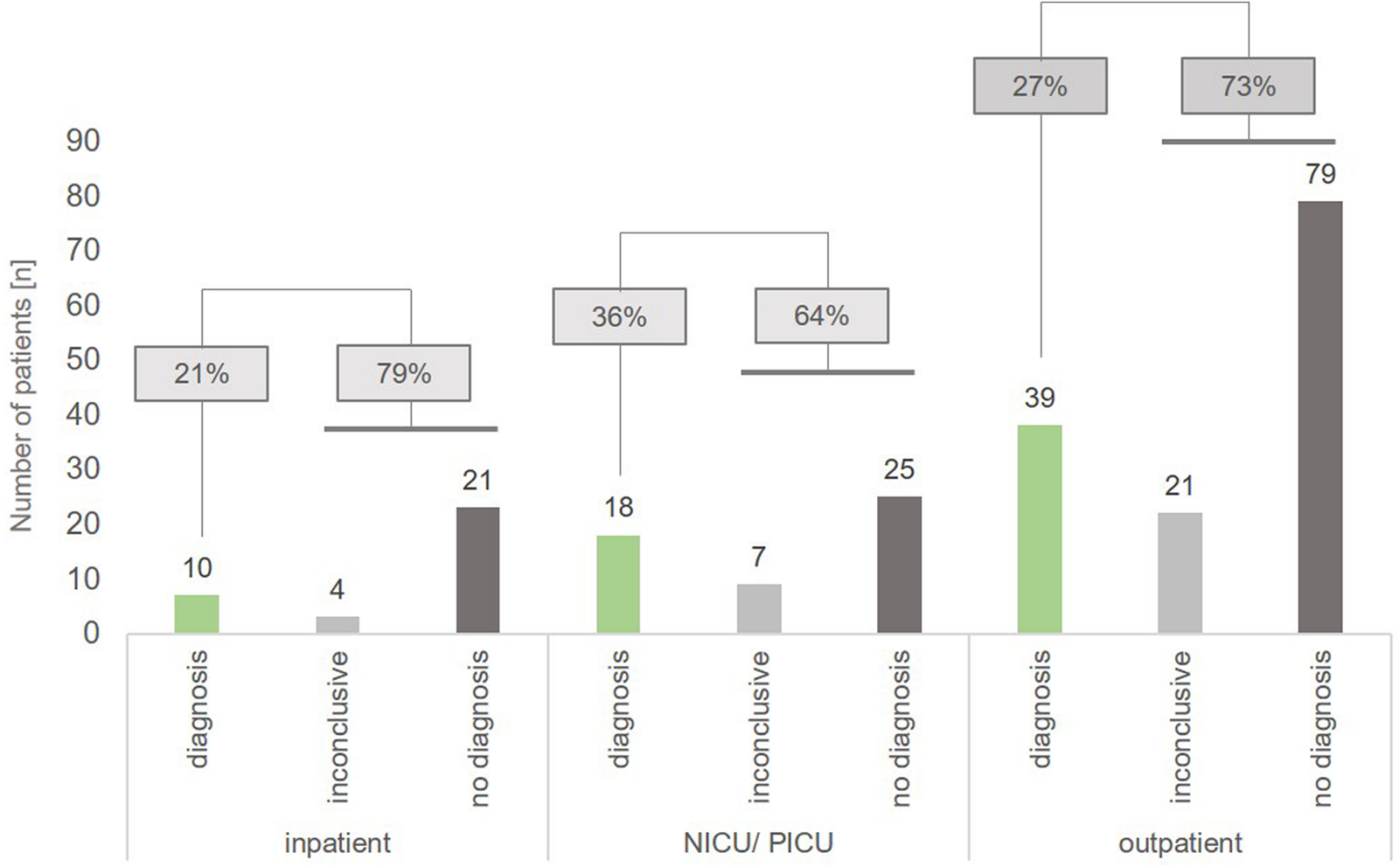
Diagnostic yield in the different sub-cohorts inpatient, NICU/PICU and outpatient. For inpatients in normal care units, a genetic diagnosis (class 4 and 5 variants) was achieved in 21% of patients (*n* = 10), while ICU patients had a diagnosic rate of 36% (*n* = 18). In outpatients, a genetic diagnosis was obtained in 27% of cases (*n* = 39).

We also analyzed the diagnostic yield in different sub-cohorts (NICU/PICU, inpatient, outpatient): tWES identified causative variants indicating a monogenetic disorder in 36% (*n* = 18) of patients hospitalized on a NICU or PICU (*n* = 50), while 64% of these patients carried VUS (inconclusive, *n* = 7) or had a negative result (no diagnosis, *n* = 25). In comparison, we achieved a diagnostic yield of 21% (*n* = 10) in non-critically ill inpatients and 27% (*n* = 39) in outpatients, respectively. 79% of inpatients and 73% of outpatient children had an inconclusive or negative result (Figure 1).

**Figure 2:**
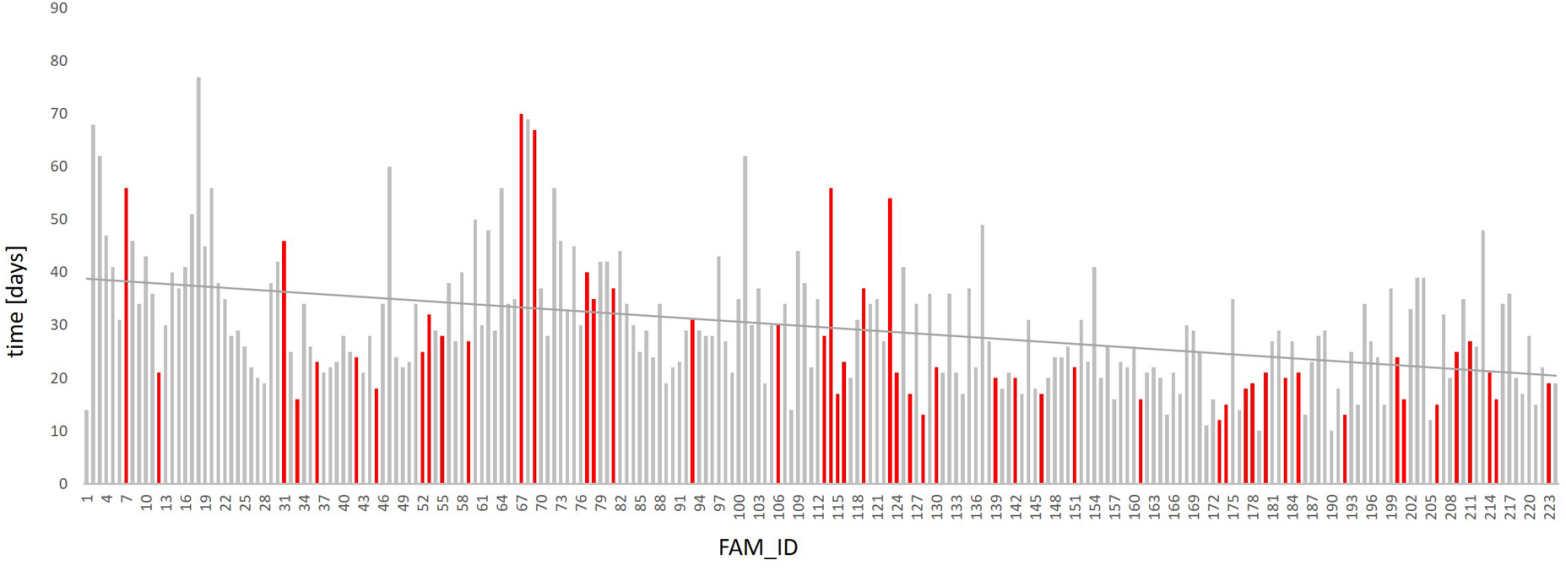
Time required for genetic analysis. The reported time required for DNA preparation, NGS and preparation of scientific report of all 224 analyzed patients is given. The reporting time for the 224 trio analyses have been continuously dropping since 2019. While the turnover time still averaged at 41 days in 2019 (FAM1-FAM14), it was reduced to 34 days in 2020 (FAM15-FAM126) and to 23 days in 2021 (FAM127-FAM224). The family IDs of the individual patients are listed (FAM_ID) (*X*-axis). Each bar represents one patient; red bars refer to patients admitted to NICU/PICU; the time required for genetic analysis is given in days (*Y*-axis, time). The linear trend line is shown in red.

The overall mean time to report the results of the molecular analysis was 30 days but the average duration of evaluation was reduced from 41 days in 2019 (FAM01-FAM14), and 34 days in 2020 (FAM15-FAM126) to 23 days in 2021 (FAM127-FAM224), reflecting a TAT reduction of almost 44% (Figure 2). This is mainly explained by expertise acquired over time in the analysis of the data and a better understanding of the clinical interpretation of the results. Furthermore, due to an increasing demand of diagnostic exome sequencing and thus a higher sample volume per time, the TAT could be reduced because the required minimum batch size for economic sequencing runs could be utilized faster. The overall median time to report the results of the molecular analysis especially in children treated at ICU was 39 days in 2019, 33 days in 2020 and 19 days in 2021. This even corresponds to a reduction of more than 52%. No relevant difference in TAT was obtained when comparing reports with and without genetic diagnosis or with in inconclusive result (data not shown).

### 3.2. Genetic spectrum of diagnostic variants

We identified causative variants in 54 genes as classified according to ACMG guidelines. Table 2 shows the diagnoses per disease group. Three molecular diagnoses were made by submitting novel candidate genes with highly suspicious variants to GeneMatcher (17). Matching to multiple other cases reported in GeneMatcher enabled establishment of novel causative gene–disease associations, namely *TMEM222* (FAM_60), *ZNFX1* (FAM_75) and *GAD1* (FAM_116). Biallelic p/lp variants in *TMEM222* cause an autosomal recessive neurodevelopmental disorder (25), biallelic p/lp variants in *ZNFX1* lead to multisystem inflammation and susceptibility to viral infections (26) and biallelic p/lp variants in *GAD1* are causal for an early-infantile onset epilepsy and developmental delay (27, 28). GeneMatcher matches were obtained for five additional genes; these genes are currently subject of further investigation (data not shown). Variants in other five genes were initially reported as VUS but were reclassified as p/lp variants following segregation analysis of further family members [*FTDC* (FAM149); *WWOX*, (FAM31); *SLC52A2* (FAM126)], allowing to apply the ACMG criteria PP1 (cosegregation with disease in multiple affected family members in a gene definitively known to cause the disease) or PS3 (well established *in vitro* or *in vivo* functional studies supportive of a damaging effect on the gene or gene product). For two genes, functional studies that were published in the meantime allowed the use of PS3 [*XIAP* (FAM44) (29); *STAT3* (FAM72)] (30), leading to a reclassification. Detailed information about identified variants are given in Supplementary Tables S1, S2. Ten patients (15% of all diagnosed patients) with a monogenetic diagnosis [immunodeficiency (*n* = 2), hepatopathy (*n* = 1), neurology (*n* = 1) and syndromic disorder (*n* = 6)] had large CNVs that were detected via WES (Table 3).

**Table 2:** Genes, in which pathogenic and likely pathogenic variants were identified by tWES in our cohort, categorized in 12 disease groups.

**Cardiology**	**Nephropathy**	**Syndromology**
*MYH7*	*PKD1*	*ARID2*
*SCN5A*	**Pulmology**	*CHD7*
**Immunodeficiency**	*NEK10*	*EYA1*
*AICDA*	*SPAG1*	*KDM6A*
*ATM*	**Neurology**	*KMT2D*
*BCL11B*	*ARID1A*	*LRSAM1*
*CREBBP*	*ARX*	*MECP2*
*DOCK8*	*DMD*	*NALCN*
*IL2RG*	*GAD1*	*NFIX*
*PIK3R1*	*KCNQ2*	*PACS1*
*PRF1*	*PCDH19*	*SLC52A2*
*RANBP2*	*PTRH2*	*TRIO*
*PSTPIP1*	*SCN2A*	*TRNT1*
*SATB1*	*SCN3A*	*TWIST1*
*STAT3*	*SORD*	**Metabolic disorder**
*XIAP*	*TMEM222*	*CA5A*
*ZNFX1*	*WWOX*	*FTCD*
**Dermatology**	**Myopathy**	*MOCS2*
*FECH*	*DYNC1H1*	*SERPINA1*
**Hepatopathy**	*PHKA1*	**Mitochondriopathy**
*IFIH1*		*PDHX*

**Table 3:** Copy number variants that were identified by tWES in this study.

Disease group	FAM ID	Nomenclature (HGVS)
Immunodeficiency	FAM100	UPD 11q and *de novo* CBL:c.1151G > A p.(Cys384Tyr) heterozygous
	FAM195	NC_0000023.10(NM_032121.5):g. (77096838_77109377)_(77126442_77130906)del (MAGT1) hemizygous
Hepatopathy	FAM20	NC_000023.10:g.(152957440_152967235)del (SLC6A8 and BCAP31) hemizygous
Neurology	FAM18	NC_000002.11:g.(30144432_30369807)_(32774548_32800223)del (incl. SPAST) heterozygous
Syndromology	FAM42	NC_000023.10:g(7137748_7170270)_(13061928_13336968)del (incl. HCCS; MIDAS-syndrome) heterozygous
	FAM51	NC_000022.11:g.(18659604_18893867)_(21386121_21562409)del (22q11 deletion syndrome) heterozygous
	FAM73	NC_000012.11(NM_003482.3):c.(2797 + 1_2798-1)_(4418 + 1_4419-1)del (KMT2D) heterozygous
	FAM174	NC_000023.11:g.(14883652_14891790)_(14937951_15262628)del (FANCB Promotor—VACTERL-H) hemizygous
	FAM183	NC_000001.11:g.(1418014_1417497)_(1453175_1454280)del p.?; ATAD3A:c. 158C > T p.(Thr53Ile) (Harel-Yoon-syndrom) compound heterozygous
	FAM210	NC_000001.10:g.(16360174_16370967)_(16383431_16384911)del (CLCNKB; Bartter-syndrome Typ 3) homozygous

Among the 67 patients with confirmed molecular diagnosis, the mode of inheritance of the identified hereditary diseases was autosomal dominant in 52% (*n* = 35), autosomal recessive in 34% (*n* = 23) and X-linked in 14% (*n* = 9). *De novo* variants accounted for 46% (*n* = 31) of all p/lp variants, whereas inherited variants accounted for 54% (*n* = 36). 6 out of 9 X-linked variants were inherited from the unaffected mother (Supplementary Tables S1, S2).

### 3.3. Re-evaluation of exome data

To test the validity and usability of trio analyses, we performed new variant annotation and re-evaluated all 224 tWES data at least 1 year after the last reporting. In none of the trio analyses, a new diagnosis was established by re-evaluation. Also, no additional variants could be identified, that were suitable for a GeneMatcher upload.

### 3.4. Secondary findings

In three parents of the analyzed 224 paediatric patients (1.3% of all tested families), we identified a p/lp variant which was not associated with the medical indication for testing and were disclosed to the families as secondary findings. This involves variants in the genes *BRCA1* (*n* = 1; FAM215) and *BRCA2* (*n* = 2; FAM91; FAM209), which are associated with an increased risk of hereditary breast and ovarian cancer (HBOC).

## 4. Conclusion

Positive as well as negative genetic test results in patients with suspected rare genetic diseases can lead to adjustment of treatment and facilitate accurate and evidence-based decisions in affected families (9). In this diagnostic setting, analysing 224 paediatric patients with suspected rare mendelian disorders, we provided a diagnosis in 67 (30%) of all analysed children, and in 18 (36%) children treated in ICUs using tWES. P/lp variants could be identified in 54 different genes, including three genes that were previously not associated with a clinical condition. 10 children (15%) with a genetic diagnosis had large copy number variations that were detected via tWES. To the best of our knowledge, this is the largest study published to date on paediatric patients with a broad spectrum of suspected genetic disorders analysed by tWES.

The American College of Medical Genomics (ACMG) recommends re-evaluation of sequencing data every 2 years (31). Some studies report that periodic data re-evaluation can be a valuable approach to identify additional variants and increases the diagnostic yield up to 10%–12% (32–34). Interestingly, in none of our trio analyses a new diagnosis was established by re-evaluation the data at least 1 year after the last reporting. This underlines the particular effectiveness and reliability of a trio-analysis at the time of diagnostic reporting. The combination of a thorough analysis of the sequencing data (and CNV data), including literature search of candidate genes and the use of GeneMatcher seems to be very effective. In this study, a diagnosis was established in three cases using GeneMatcher (25, 26, 28) and another five cases are currently subject of further analysis after identification of further patients via GeneMatcher (data not shown). This corresponds to a rate of 1.9% (3/157) of definitely and an additional 3.2% (5/157) of potentially solved cases using GeneMatcher in all analysed 157 tWES families with an initially negative or inconclusive diagnostic report.

A few studies are available that evaluate the effectiveness of different NGS methods in pediatric patients. Some studies describe the diagnostic impact of WES in children with neurodevelopmental disorder (35). All of these studies differ slightly in patient's population, size and diagnostic yield. Since 2015, reports have also been published about the diagnostic value of WGS and the comparison to WES in different disease groups (14, 36–40). A few published data are available describing the diagnostic yield of tWES or tWGS (41–46), but to the best of our knowledge there is only one detailed description of such a large cohort analysed via tWES in a diagnostic setting (47).

In our cohort, p/lp *de novo* variants of 15 patients would have been classified as VUS in singleton WES analysis according to the current ACMG guidelines. This corresponds to a rate of 22% (15/67) of all diagnosed patients. Particularly, in patients with early-onset and severe complex phenotypes, a fast genetic diagnosis could be essential for clinical decisions, which is facilitated by parental genetic information. Five out of 15 infants with *de novo* variants were critically ill and treated at NICU/PICU at the time of the genetic analysis, therefore potentially benefitting from a fast diagnosis.

However, all *de novo* variants classified in this cohort as p/lp would also have been highly suspicious candidates in singleton analysis. This raises the question of whether a singleton analysis is sufficient in patients who do not need to be diagnosed particularly quickly. tWES avoids the delay of confirmatory testing for phasing variants in recessive disorders and the determination of inheritance which can upgrade or downgrade their pathogenicity classification according to ACMG guidelines, thereby increasing diagnostic yield. Finding the right approach for each patient is therefore not self-evident.

Unfortunately, only a few studies have been published so far that provide a comparative analysis of the diagnostic efficacy and the costs between singleton and trio analyses (48, 49). In 2019, Tan et al. analysed 30 cases as tWES and singleton WES in parallel (48). The authors concluded that tWES does not significantly increase diagnostic yield compared to single WES, but that tWES approximately halves the time required for prioritization and curation of variants compared to singleton analysis. In addition, they pointed out that analysis of tWES data reduces the cost by selecting fewer variants for curation, avoiding reporting of variants with unclear significance, and eliminates the need for time consuming sequential parental sequencing. Thus, at least in critically ill patients, where a timely genetic diagnosis has the potential to crucial changes in medical management, tWES should be considered as first-line diagnostics.

In our study, the inclusion of exome-based CNV analysis made tWES a powerful tool for diagnosing genetic diseases in additional ten patients just as other studies have reported an increase in detection rate after inclusion of CNV analysis (50). The rate of “secondary findings” in our cohort was 1.3% for the above mentioned 12 genes (in minors) and 17 genes (in adults). The commonly known rate for the detection of secondary findings in clinical exome sequencing is 1%–3%, mainly depending on how many genes were included in secondary analysis. The ACMG initially defined a list of 59 genes associated with secondary findings that should be reported in patients undergoing clinical exome sequencing (51). This gene list was increased to 73 genes (v3.0) (52) and currently includes 78 genes (v3.1) (53).

In summary, our study shows that trio-based exome sequencing in routine diagnostics is effective to obtain a fast genetic diagnosis in paediatric patients. A holistic approach including comprehensive variant assessment of suspected variants, evaluation of CNV data and the use of GeneMatcher maximises the diagnostic output. It is desirable to offer trio sequencing to all patients with suspected monogenetic disease, but especially to critically ill patients, even outside studies within regular healthcare service to minimize the “diagnostic odyssey”.

## Data Availability

The datasets for this article are not publicly available due to concerns regarding participant/patient anonymity. Requests to access the datasets should be directed to the corresponding author.
